# Supplementary material for: Are carbon emissions trading and green financial instruments synergistic? -Comprehensive quantitative research based on content analysis
Source: PLoS One. 2024 Mar 7;19(3):e0298601. doi: 10.1371/journal.pone.0298601 (PMC10919601; doi:10.1371/journal.pone.0298601)
Supplement: S1 Data — (ZIP) [file pone.0298601.s001.zip › Data/地级市Z_F工作报告.zip!/║■▒▒/╩«╤▀╩╨/└┤╫╘╣1⁄2╓┌║┼í╛┬φ┐╦╩2╛▌═°í┐_2013.docx]

各位代表：

现在，我代表市人民政府向大会作工作报告，请予审议，并请各位政协委员和其他列席会议的同志提出意见。

一、2012年工作回顾

2012年是特别困难的一年，也是全市迎难而上、大有作为的一年。面对国内外经济环境复杂多变、商用车市场持续低迷、百年不遇特大暴雨灾害袭击等严峻挑战，在市委的坚强领导下，全市上下和衷共济、共克时艰，取得了经济社会发展的新成就，实现了保民生、保增长、保发展、保稳定目标。预计全年实现生产总值950亿元，同比增长11%；财政总收入120.7亿元，增长13%，其中地方公共财政预算收入76.9亿元，增长15.3%；全社会固定资产投资680亿元，增长30%；社会消费品零售总额430亿元，增长15.6%；外贸出口3.1亿美元，增长20.7%；实际利用外资1.3亿美元，增长22.7%；城镇居民人均可支配收入1.6万元、农民人均纯收入4570元，均增长13%；万元生产总值综合能耗下降3.5%；城镇登记失业率控制在4.5%以内；人口自然增长率控制在7‰以内；居民消费价格总指数累计上涨3%。除工业增加值外，完成了市四届人大二次会议确定的主要目标任务。

（一）国民经济保持平稳增长

——加快发展现代农业，农村经济提速增效。以“三化”协调发展和“一统三分”统揽“三农”工作。精心培育特色农业，突破性发展农产品加工业，扎实推进新农村建设，着力夯实农业基础，奋力组织抗灾救灾，深入开展扶贫攻坚，努力提高农民收入，农村经济发展态势良好。完成农业增加值122亿元，增长25.9%，农民人均纯收入净增526元。粮食实现“九连增”。新建特色产业基地28.5万亩、标准化畜禽养殖小区120个；17个农产品获“三品一标”认证；新增市级以上龙头企业44家，农产品加工产值达120亿元，增长42.9%；新增专业合作组织138家。新建农村新社区25个、村级综合服务社300家。改造新修县乡村道路1348公里，除险加固病险水库21座，整治塘堰7056口，完成水土流失治理120平方公里，解决25万人饮水安全问题，十堰市荣获全省水利建设“大江杯”优秀组织单位奖。扶贫搬迁3048户，减少贫困人口8.5万；改造危房1.6万户，完成65个重点村整村建设任务，秦巴山片区扶贫开发工作全面启动。大力开展灾后恢复重建工作，实施水库修复工程33项，加固整修塘堰336座，修复基本农田4.9万亩，修建倒塌民房10059间。

——积极应对巨大挑战，工业经济稳中有进。全力服务东风公司，加快工业项目建设，推进工业结构调整，工业经济在异常困难的情况下实现平稳增长。完成规模以上工业增加值390亿元，增长5%。东风装备新工厂、东风特汽、三环专汽、同创汽车同步器、川路汽车、丹江铝业超薄铝箔坯等项目竣工投产；国家汽车零部件产品质量监督检验中心、房县精细磷产品产业园等项目开工建设。大力推进“满园工程”，引进工业项目226个、资金113.7亿元，徐工金三角随车吊、苏州朗格电动车、远大新型环保材料、合肥常青机械等项目落户十堰市。继续推进“双亿工程”和“中小企业成长工程”，新增规模以上企业83家。十堰市被科技部授予“国家商用车零部件高新技术产业化基地”称号，武汉国家生物产业基地十堰生物产业园创建成功，武当酒业在天津股权交易所挂牌上市。大力开展非法拼装车“清剿战役”，净化了汽车市场秩序。驰田金钢荣膺“中国驰名商标”。县域工业快速增长，完成工业总产值393亿元，增长22%。9家开发区发展势头良好，完成产值570亿元，增长20%。

——着力优化产业结构，第三产业乘势快上。推进旅游产业加快发展，促进商贸流通业扩大规模、现代服务业取得突破。第三产业占GDP比重达37.4%，提高1.9个百分点。全年接待国内外游客2410万人次，实现旅游收入166亿元，分别增长29.3%和39.2%。旅游项目建设多点发力，完成投资60亿元，增长95%，太极湖生态文化旅游区一期、武当山玉虚宫和五龙宫大殿修复竣工，郧阳岛、天河旅游景区建设加快推进；新增4A级景区4家、旅行社1家。房县荣获“湖北旅游强县”称号，竹溪偏头山森林公园晋升为国家级森林公园，武当道茶文化旅游山庄成为国家级休闲农业与乡村旅游示范点，武当山至神农架、三峡旅游直通车正式开通。商贸流通业快速增长，万村千乡、家电下乡工程深入推进，新（改）建农家店211家；家电下乡销售8.6亿元，兑付补贴9907.4万元，十堰市被纳入“全国家政服务体系建设试点城市”；一批物流项目正在开展前期工作，寿康永乐长岭物流配送中心竣工；信息、保险、中介等新兴服务业迅速发展；银行机构各项存款余额1216.5亿元，比年初增加220亿元，增长20%，各项贷款余额594.9亿元，比年初增加110亿元，增长21%。

——千方百计扩大投资，发展后劲不断增强。深入开展“重大项目建设年”活动，竭尽全力策划、争取、引进项目，精心组织、热忱服务项目，固定资产投资保持高速增长。投资结构进一步优化，重点项目进展顺利，年初确定的29个省级、200个市级重大项目全部开工。十房、谷竹、十白、郧十四条高速公路累计完成投资230亿元，占总投资的72%；丹江口环库公路启动建设，丹土一级路路基全线贯通；机场大道进展顺利。潘口、小漩、三里坪水电站并网发电，龙背湾水电站实现截流，孤山水电站前期工作全面铺开；京能热电项目已报国家发改委待批。一批产业园区开工建设，共开山整地1.8万亩、新增建设用地2.6万亩，十堰农产品加工工业园、茅箭循环经济产业园完成了土地平整。丹江口库区“移土培肥”工程启动实施。十宜铁路、武汉至十堰城际高铁项目进入省部合作规划。

（二）“一城两带”建设有了重大进展

——中心城区建设管理加强。城市总体规划、中岳路片区等8项城市控详规编制完成，“数字十堰”技术方案通过专家评审，区域性中心城市规划修编、生态滨江新区规划编制全面启动。郧十一级路、东环路、建设大道二期、北京路延长线、郧阳汉江大桥建成通车，发展大道、中岳路、林荫大道二号线一期工程加快推进，风神大道、第三水厂取水口工程开工建设；天然气城网工程、西部污水处理厂建设竣工，城中村改造和棚户区改造稳步推进，50条背街小巷、6处交通堵点和公园路改造完成，车城广场基本竣工；城区“三违”整治集中展开；创卫和城市管理成果不断巩固；茅箭区成功争创全国国土资源节约集约模范县（市），十堰市集约节约利用土地模式受到国土资源部高度赞誉。

——“两带”建设全面展开。坚持以优势产业打造、城镇村庄扩容、基础设施完善、生态环境改善、体制机制创新、公共服务优化、精神文明提升为重点，全面加快竹房城镇带建设。共实施交通、水利、城镇、土地整理等项目335个、投资近20亿元；发展特色产业基地9.6万亩，改造村庄35个，新建“三位一体”服务中心66个、新进城镇农户2.3万户8.9万人，城镇带GDP增长21.9%、人均纯收入增长14%、城镇化率达到54.9%；房县“三化”协调发展示范区建设全面启动，竹山县、竹溪县抢抓城镇带建设机遇，立足特色，争先进位，取得了发展新成效。坚持把新农村建设、扶贫开发、移民安置、小流域治理、土地整治结合起来，全面推进汉江生态经济带建设，扎实开展示范乡镇、示范村建设，启动郧县生态滨江新区规划和建设，《丹江口库区及上游水污染防治和水土保持“十二五”规划》、《丹江口库区及上游地区经济社会发展规划》获得国务院批复并逐步组织实施，库区生态经济发展步伐加快。

（三）改革开放实现新的突破

——各项改革深入推进。深入推进经济、投融资、社会事业、民生保障、行政管理等体制机制改革。税收“营改增”试点顺利展开；成功发行12亿元城投债券，通过信托、票据等方式融资11.5亿元，组建7家小额信贷公司，农村信用社改革全面完成，交通银行入驻十堰，十堰市连续七年获得“金融信用市”殊荣；农村土地承包关系进一步规范完善，流转土地27万亩；继续推进医疗卫生体制改革，丹江口市、竹山县被纳入全国县级公立医院综合改革试点；城管综合执法体制、市级食品安全监管体制、事业单位绩效工资制度改革推进到位；行政审批“三集中、三到位”和“四减五制六公开”改革扎实推进，减少审批事项291项。

——对外开放成效显著。实施开放先导战略，狠抓招商引资。重新组建招商局，成立九大产业招商办公室，出台招商引资奖惩考核办法，大力开展全员招商、全域招商、全天候招商、全过程招商，组织和举办了湖北十堰——浙江（长三角地区）经贸洽谈会、台湾·十堰经贸文化交流活动、武当大兴600年·湖北十堰经贸合作洽谈会、第十二届全国村长论坛·十堰投资推介说明会等重大招商活动。引进市外境内项目371个，到位资金170.3亿元，增长37.5%。与中亚地区、大洋洲外经合作实现突破，境外投资企业达到7家。组织参与了华创会、湖北·韩国经贸文化交流活动，接待重要外宾、侨商侨领16批次，对外合作领域进一步拓展。

（四）社会事业得到持续加强

优先发展教育事业，大力推进科技进步，努力推动文化繁荣，着力保障人民健康，整体提升计生水平，社会事业全面协调发展。新认定高新技术企业30家，申请专利1700件，创建国家知识产权示范城市通过验收，被省委、省政府授予“科技创新先进市”称号。改扩建幼儿园170所，改造中小学危房12万平方米，32万人次享受学生资助政策，义务教育发展趋向均衡，扎实推进素质教育，教育质量稳步提升，湖北汽车工业学院和湖北医药学院获硕士学位授权。文体活动丰富多彩，《我的汉水家园》等12件作品在第一届湖北艺术节上获奖，新建农民体育健身工程120个，广播电视混合覆盖率达97.7%。政府举办的基层医疗卫生机构100%配备基本药物；新型农村合作医疗参合率达97.8%，政策范围内住院报销比例达75.3%；农民看病就医“一卡通”全面实施；创建国家级临床重点专科4个、省级17个，茅箭区成为国家级慢性病综合防控示范区。低生育水平保持稳定，出生人口性别比下降1.6个单位值，人口和计划生育整体水平明显提升。创建国家环保模范城规划通过国、省评审；完成造林32.6万亩，森林覆盖率达55%；完成155个村庄环境连片整治示范工作，创建国家级生态乡镇3个；建成并投入运行污水处理厂11座、垃圾处理场6个；十堰市被授予全国环境规划十佳城市。

（五）民生保障取得良好成效

坚持以人为本，突出执政为民，大力保障和改善民生。民生支出达146.6亿元，占地方公共财政预算支出的71.5%。城镇居民人均可支配收入和农民人均纯收入大幅提高，增幅分别超GDP 2%。新增城镇就业3.2万人；五项社会保险和住房公积金参保新增9.2万人次。市社会福利服务中心和居家养老服务中心建成使用，改扩建农村福利院30所，城乡高龄补贴制度全面建立，城乡居民养老体系实现全覆盖，发放城乡低保资金4.6亿元，扩大五保供养对象1.1万人。开工建设保障性住房20195套，发放住房补贴3548户。优先发展公交事业，新开辟公交线路5条，十（堰）郧（县）城际公交开通。“数字化城管”和“网格化社区”建设扎实推进，一格一员、一区一警、一街一队新型管理格局初步形成。市长公开电话“民生直通车”作用进一步发挥，办理解决群众反映问题5.2万件。积极实施信访积案化解攻坚，按期办结率达97.5%。深入开展打黑除恶、治安攻势等专项行动，群众安全感、治安满意度分别达93.8%和93.7%。加强安全生产工作，安全事故数量和死亡人数双下降。坚持整顿和规范市场经济秩序，食品、产品质量安全水平不断提高。着力稳控物价，居民消费价格总水平回落3个百分点。十件实事全面超额完成。

（六）精神文明和民主法制建设收获丰硕成果

深入开展文明创建，大力弘扬时代新风，扎实推进依法治市，切实加强政府自身建设。涌现了“最美姑娘”周丽、“最美搬运工”甘龙宝、“最美乡村教师”邹桂芬、“最美电工”史克军、李琼、陈品东三位烈士等一大批先进典型，竹山县“十星级文明户”创建工作再次得到中央文明委充分肯定，出租车行业文明创建经验在全国推广，市供销社被授予“全国供销社系统先进集体”称号，顺利通过全国城市文明程度指数暗访测评，十堰市蝉联全国双拥模范城“六连冠”和全省双拥模范城“七连冠”，国防动员、民兵预备役工作取得新成效。自觉接受人大、政协监督，定期向人大报告工作、向政协通报情况，办理人大代表议案、意见、建议181件，办理政协委员提案353件，办结满意率均达100%；“六五”普法和法治十堰建设深入推进，十堰市荣获“全国法治城市建设先进单位”和“省委、省政府‘六五’普法宣传先进单位”称号。扎实开展民主评议政风行风活动，机关作风进一步转变，政府廉洁自律各项规定进一步落实。审计促进重大政策落实和保障财政资金安全作用充分发挥，财政管理工作进一步加强并受到省高度评价。第八届村委会换届选举工作全面完成，村务公开覆盖率达100%，竹山县被命名为“全国村务公开民主管理示范单位”。

全市统计、民族宗教、对台、气象、水文、地震、人防、通信、残联、社科、档案、地方志等工作也都取得新的成绩。

各位代表！刚刚过去的一年，是十堰市发展史上极不平凡的一年。这一年，我们抢抓机遇，深度谋划，成功争取省委、省政府大力支持，十堰市区域性中心城市建设上升为省级战略，成为全省“一元多层次”战略体系的重要组成部分！这一年，我们不辱使命，勇于担当，圆满完成了南水北调丹江口库区18万移民搬迁安置，顺利实现了省委、省政府提出的“四年任务，两年基本完成、三年彻底扫尾”的工作目标，创造了世界移民史上的伟大奇迹！这一年，我们万众一心，众志成城，合力战胜了百年不遇的特大暴雨灾害，铸就了不畏艰险、自强不息的伟大抗洪精神！这一年，我们齐心协力，积极作为，成功举办了武当大兴600年盛典、第十二届全国村长论坛等大型活动，在国内外产生了重大影响；成功争取武当山机场顺利通过国务院、中央军委批准立项并开工建设，圆了十堰人民28年的机场梦；成功争取十堰经济开发区晋升为国家级经济技术开发区，为十堰市又增添一块“金字招牌”，增强了十堰发展的吸引力和竞争力。这一系列喜事、大事、难事的办成办好，必将对十堰市未来产生重大而深远的影响，必将激励我们立足新起点、构筑新优势、再创新辉煌！#PAGE_BREAK#

各位代表！过去一年，成绩来之不易。这是省委、省政府和市委正确领导的结果，是市人大、市政协监督支持的结果，是全市人民奋力拼搏的结果。在此，我代表市政府向所有参与、支持、关心十堰发展的各界人士，表示衷心的感谢并致以崇高的敬意！

在总结成绩的同时，我们也清醒地认识到，经济社会发展中还存在许多问题和不足，主要是：发展不快、发展不够，特别是县域经济发展滞后是十堰市最大的问题，总量规模和综合实力亟待扩大和增强；农业抗御自然灾害和市场风险能力脆弱，工业结构单一，服务业发展不快，结构调整任务艰巨，能源、资金、土地等要素制约突出，生态环境保护压力加大，深化改革开放和加快转变经济发展方式十分紧迫；城乡居民收入水平不高，基本公共服务体系不健全，民生改善与人民群众的期盼还不相适应，尤其是贫困人口较多、贫困面较广，扶贫攻坚任务十分艰巨；经济发展环境不优，少数部门和个人服务不力、创新不够、执行力不强，公务员队伍的作风、能力、素质有待进一步转变和提高等。对此我们将高度重视，采取有效措施，认真加以解决！

二、2013年工作安排

2013年是贯彻党的十八大精神的开局之年，是实施“十二五”规划承前启后的关键之年，是为全面建成小康社会奠定坚实基础的重要之年。我们面临一系列重大机遇：一是发展阶段、发展格局变化带来新机遇。世界经济深度转型调整倒逼国内经济走扩大内需、提高创新和转变经济发展方式的转型之路，产业梯度转移加速，有利于后发地区加快发展、实现赶超。二是国家和省实施一系列重大战略带来巨大的政策利好。国家继续实施积极的财政政策和稳健的货币政策，把扩大内需作为重要战略基点，大力推进城镇化和生态文明建设、加快推进秦巴山片区扶贫开发、深入实施丹江口库区“两规划一方案”，省委、省政府实施“一元多层次”战略、支持十堰市建设区域性中心城市，有利于十堰市获得更大的支持。三是近年来十堰市经济社会发展已有较好基础，东风公司推进“大商用车”战略，将为未来发展创造更好条件。

与此同时，我们面临的压力和挑战也不可低估：一是宏观环境极其复杂，尤其是经济增长下行矛盾依然突出；二是加快发展、加快转型双重任务更为紧迫，统筹兼顾难度更大；三是周边地区竞相发展势头强劲，区域竞争日趋激烈。十堰市与全面建成小康社会还存在较大差距。为此，我们必须强化机遇意识，寻机而发，谋势而动，乘势而上，必须有战胜困难的思想准备，必须有夺取胜利的坚定信心，努力实现发展的新突破。

今年政府工作的总体要求是：以党的十八大和中央、省经济工作会议精神为指导，坚持主题主线，调整优化经济结构，奋力推进城乡统筹，大力推动改革开放，努力保障改善民生，全面实施区域性中心城市战略，加快构建“一核多支点”新格局，竞进提质、开拓创新，确保经济运行速度、质量、效益明显高于上年，确保南水北调中线工程后续工作圆满完成，确保扶贫攻坚、民生改善取得重大进展，确保社会大局和谐稳定，实现“六增”(增速度、增效益、增投资、增项目、增后劲、增收入)目标，开创全市转型发展、跨越发展新局面。

经济社会发展的主要目标是：地区生产总值增长12%；地方公共财政预算收入增长15%（剔除南水北调工程一次性收入后，按可比口径计算）；固定资产投资增长30%；社会消费品零售总额增长16%；规模以上工业增加值增长12%；实际利用外资增长10%，外贸出口增长10%；城镇居民人均可支配收入和农民人均纯收入均增长13%；城镇登记失业率控制在4.5%以内；居民消费价格指数、单位生产总值能耗下降、主要污染物排放量控制在省定目标以内。

需要说明的是，上述目标只是底线目标，在实际工作中，我们将本着“跳起来摘桃子”的精神，力争实现更好更快发展。重点做好以下工作：

（一）以提高经济运行速度、质量、效益为中心，奋力推动经济发展

——做大总量，优化结构，促进工业提质增效。牢固确立工业兴市意识，高扬工业化龙头，坚持扩大总量与优化结构并重，提升传统优势产业与壮大现代新兴产业并举，培育产业与做强企业齐抓，加快提升工业整体素质，力争工业产值达到1500亿元、增加值达到440亿元，新增规模企业100家以上。一是壮大工业块头，实现工业快速增长。实施三年工业倍增计划，分产业、行业、企业进行“点对点”支持帮助；加大工业投资力度，加快工业项目建设，大力培植一批投入和产出过百亿、十亿、亿元的重点产业和骨干企业；着力扶持中小企业、小微企业发展壮大；全力培育千亿级汽车工业、千亿级县域工业和千亿级园区工业。二是下真功夫优化工业结构，形成一主多元、多级支撑的产业格局。在做强做大汽车产业、落实《政企共建百万量级汽车城合作框架协议》、助推东风公司提能增效并辐射带动地方工业不断扩张的同时，进一步壮大能源、农产品加工、生物医药、食品饮料业，努力建成3至5个过百亿的特色工业。大力发展县域工业，县域工业比重达30%以上，“十二五”末达35%以上。调整汽车产业内部结构，地方汽车工业要与东风配套发展、互补发展、错位发展，致力发展专用车和关键零部件，力争专用车突破15万辆。大力调整产品结构，支持企业开展技术创新，增强质量品牌意识，研发生产科技含量高、市场竞争力强的产品。三是用硬措施推进园区建设。进一步拓展工业发展空间，加快园区土地整理步伐，完善基础设施配套建设，使项目及时落地。各县市区都要集中精力建好具有一定规模的工业园区。加快以国家经济技术开发区、国家新型工业化示范基地和国家生物产业基地十堰产业园为龙头的园区建设，深化国家商用车零部件产业集群创新示范区和国产数控装备应用示范区创建，抓紧创建国家高新技术产业开发区。新增高新技术企业30家。四是培育优秀企业家队伍。尊重企业家、服务企业家，建立交流平台，组织学习培训活动，为企业家提升素质增强能力提供机会，造就一个志向高远、奋发向上、诚信经营、扎实推进企业提质增效加快发展的企业家群体，涌现一批为十堰发展贡献力量的栋梁。

——突破加工，创新机制，提高农业产出能力。强化农业基础地位。坚持用工业化理念谋划农业，用市场化机制激活农业，用信息化手段武装农业，用标准化生产提升农业，用合作化组织整合农业，促进农业跨越发展。一是大力发展特色农业。深入推进“四百万”工程，新建特色基地25万亩，新增蔬菜基地1万亩、山羊6万只、标准化养殖小区100个，创建郧西、房县现代山羊养殖示范区，新增农业“三品一标”认证20个以上，支持“武当道茶”争创“中国驰名商标”、房县黑木耳争创首批全国百强区域公共品牌。二是提高农业工业化水平。强力发展农产品加工业，深入实施“四个一批”工程，加快推进十堰（长岭）、丹江口市等农产品加工园区建设，引进和培育加工龙头，启动龙头企业“双十”工程，确保新增省级龙头企业2家，农产品加工产值过200亿元。三是增强农业产出能力。深入推进科技兴农，抓好关键技术推广，农业科技普及率达90%以上；加强信息平台建设，搞好农业信息咨询、技术服务；提高涉农从业者素质，培训农民、企业家和管理员3万人以上；大力推广农业标准化；加大基础设施投入力度，新修通村公路600公里，除险加固病险水库38座，解决人饮安全12万人；切实加强新农村建设，新建村级综合服务社300家。四是创新农业生产经营机制。充分保障农民土地权益，积极引导农民依法、自愿、有偿流转土地；提高农民组织化程度，支持联户、专业大户和家庭农场等规模化经营；发展多元服务主体，新增专业合作社100家以上；加大农民转移培训力度，转移农村劳动力2.1万人。

——激活消费，做新业态，推动第三产业繁荣发展。充分发挥现代服务业在优化产业结构、增强城市吸引力、辐射力和现代化水平的引擎作用，促进第三产业发展提速。力争实现增加值410亿元，增长13%，新增限上企业100家。一是突破性发展旅游业。以建设世界旅游名城、国际旅游目的地为目标，突出项目引领、要素完善、市场开拓，强化旅游服务，形成特色消费热点。加快推进武当山、太极湖、郧阳岛等重大项目，启动建设城区旅游商品、特色小吃、文化娱乐一条街。引进、培植旅游龙头企业，加强旅游品牌营销，打造精品旅游线路，适时筹办首届南水北调中线源头水文化旅游节；开展旅游名镇创建工作。力争全年接待游客2910万人次，增长25%；实现旅游收入209亿元，增长30%。二是加速发展商贸流通业。落实国家节能产品惠民、老旧报废汽车更新补贴、降低流通费用等政策，实施“万村千乡市场工程”，信息化改造农家店200家，建设农村流通网点400个，力争实现行政村流通网点全覆盖；推动万达广场、中商华宇商业综合体、武商集团滨江量贩店、鄂西北国际农商城等建设。三是大力发展现代服务业。加快汉江国际物流园、林安物流园等项目建设，着力将物流业发展成为重要支柱产业。紧扣产业发展和民生需求新变化、新特点，积极培育家政养老、休闲健身、节能环保产品等消费热点，加快发展电子商务、网络购物等新型消费业态，大力发展金融保险、咨询信息等服务业，不断增强消费拉动力。

——创优环境，贴心服务，培育壮大市场主体。牢固树立环境就是生产力、抓环境就是抓发展、“产业第一、企业家老大”理念，优化发展环境，大力培育各类市场主体。一是完善支持政策。出台激励促进各项产业、各类实体经济、各种不同经济成分发展的办法和措施。二是提供高效服务。强化能源、土地、资金等要素保障，确保重点企业、重点项目水、电、气和合理用地需求；积极协调银企对接，加大金融支持企业力度，引导支持企业上市，利用企业债券、股票、投资基金等方式筹集资金，扩大直接融资规模。切实帮助企业排忧解难，建立重点企业、重点项目“首席服务官”制度，开展“干部进企业、服务促发展”活动，实行“处方式“、“一对一”跟踪服务，解决企业所思、所需、所急。提高办事效率，简化办事程序，优化办事流程，争创全省审批最少城市，建立工业项目零收费制度。用心、用情、用力，竭诚服务东风公司在十堰的各项事业和国际合作，共建国际商用车之都。三是促进民营经济大发展、大提升。下决心破除民间投资的“玻璃门”、“弹簧门”等体制障碍，在民间投资进入领域方面取得新突破。落实结构性减税、信贷倾斜等优惠政策，实施全民创业工程，支持小微企业发展，培育一批有潜力的中小民营企业，加快形成企业成长梯队，促进各类市场主体增规模、上水平。

——拓宽渠道，多上项目，保持投资快速增长。充分发挥投资对经济增长的关键作用。一是多途径全力争取投资。抢抓国家扩大内需和产业转移机遇，打好南水北调和秦巴山片区扶贫攻坚两张牌，积极上争、内引、外联，大力争取国家支持，确保中央和省投资明显高于上年；努力提高金融对十堰发展的支撑能力，新增贷款规模达130亿元以上；通过招商等方式大力吸引外来投资，激活和吸纳民间投资，全社会固定资产投资突破900亿元。二是项目建设全面发力。完善领导牵头、专班推进、全程服务工作机制，促进多上项目，上大项目。着力推进重大交通项目建设，构筑立体交通网络，加快武当山机场建设，全面开工环丹江口库区旅游公路凉水河至习家店段、柳陂至五峰段建设，确保十白高速公路全线通车、谷竹高速公路分段通车、十房和郧十高速公路一期工程完工，基本完成汉江航道丹白段整治工程，完成火车站站场改造，启动十堰客运西站建设，争取武汉至十堰城际高铁早日开工、十巫高速公路立项；加快推进重点能源项目，确保京能热电工程开工，实现龙背湾水电站下闸蓄水，加快推进孤山、夹河关水电站建设；全面完成丹江口库区“移土培肥”和库底清理工程；特别突出产业项目，围绕促进工业、农业、现代服务业和社会事业加快发展，不断组织大批产业项目投入建设。三是认真筛选储备项目。对照国家产业政策，及时准确把握资金投向，提早谋划和储备项目，确保项目梯次跟进；加强项目跟踪衔接，加快推进前期工作，做到策划一批、论证一批、储备一批、申报一批、实施一批。

（二）以“一核多支点”战略为支撑，深入推进城乡统筹

立足全域规划，以城区为核心，以各县市为支点，加快发展步伐，努力形成多点支撑、多点突破的发展格局。

——加快推进“一城两带”建设，着力提高城镇化水平。以推进新型城镇化统领城乡发展，城镇化率提高2%以上。加强中心城区建设，做到精心规划、精致建设、精细管理，提高城市规划、建设、管理科学化水平。加快区域性中心城市发展战略规划、生态滨江新区发展规划和中心城区近期建设规划编制工作，完成“数字十堰”地理空间框架建设，尽快完成城区控详规全覆盖工作。加快市政基础设施建设，增强城市综合承载能力，提升完善城市功能，确保中岳路、发展大道、风神大道、机场大道、林荫大道二号线一期建成通车，推进郧阳路、京东路、林荫大道二号线二期、凤凰路、西城大道、和谐大道建设；解决一批主城区交通堵点，开通城区至武当山城际公交；加快推进三水厂建设，完成天然气对接入户工程。着力提高绿化、美化、亮化水平，建设火车站北广场和汉十高速、十白高速十堰西两个出口景观，新建一批街头游园，改造一批山地风景区，加快城中村、棚户区改造和拆迁群众安置工作；深入开展综合执法，推进“三违”整治，加强城市管理，确保在全省城管考核中取得佳绩。

深入推进竹房城镇带和汉江生态经济带建设，着力构建以县城为支撑、中心镇为节点的现代城镇体系，突出抓好6个县市城区、一批重点镇和中心村建设，大幅度提高城镇化率。加快基础设施建设，确保竹溪绕城一级公路、丹土一级公路建成通车，房县城关至军店一级公路、竹山县城至潘口一级公路基本建成；启动郧县长岭片区道路交通和生态绿地建设。实施产业带动，培植一批企业和各类经济实体，引导农民进城进镇、进入非农产业。构建科学合理的城镇格局，增强产业发展、公共服务、吸纳就业、人口集聚功能，有序推进农业转移人口市民化，采取措施，解决进城农民户籍、住房、教育、社保等问题，增强融入城镇的生活能力。

——大力发展县域经济，培育新的经济增长极。坚持“四化同步”，发展县域经济。研究激励县域经济发展政策，提出县域经济发展目标，明确县域经济发展要求，增强发展县域经济动力，不断缩小与全省的差距。根据市委、市政府的安排，切实支持郧县建设生态滨江新区、房县建设“三化”协调发展示范区，鼓励和支持丹江口市、郧西县、竹山县、竹溪县依托自身优势，找准目标定位，积极探索适合县域特点的发展路径。不断提高县域经济比重，努力把县域经济培育成新的经济增长极。

——深入实施扶贫开发，加快脱贫致富步伐。全面落实《秦巴山片区区域发展与扶贫攻坚规划(2011-2020)》，率先启动一批跨区域、打基础、管长远的重大基础设施、产业发展和民生项目，着力构建“六位一体”扶贫工作格局。深入推进丹江口市扶贫奔小康、老区连片开发、整乡推进扶贫搬迁、产业扶贫等试点示范项目，实施新一轮城区农村扶贫开发，启动整村推进重点村65个，改造农村危房1.2万户以上，扶贫搬迁3000户，培训贫困劳动力8000人，确保减少贫困人口9万人。#PAGE_BREAK#

（三）以体制机制创新为动力，不断深化改革开放

——深入推进各项改革。坚持和完善农村基本经营制度，构建和完善农村社会化服务体系。进一步深化财政四项改革，加强财政科学化精细化管理。着力推进金融创新，不断深化投融资改革，加强政府融资平台建设。巩固完善基本药物制度，全面启动县市公立医院综合改革。深入推进文化体制改革创新，加快国有经营性文化事业单位转企改制。不断创新体制机制，加速推进要素配置市场化改革。继续推进行政审批“三集中、三到位”和“四减五制六公开”改革，完善市县（市区）乡（镇）村政务服务体系。完善事业单位聘用制度，加强事业单位岗位设置常态化管理。

——强力推动招商引资。坚持把招商引资作为全部工作的重中之重，花大力气、狠下重手强力推进。进一步强化招商举措，创新招商办法，明确招商任务，落实招商政策，兑现招商奖励，组织全员招商、专班招商，提高招商水平，提升招商质效，全年招商到资资金突破200亿元。

——全面扩大对外开放。以开放为先导，加快培育开放性经济。加强十堰出入境检验检疫局、公路二类口岸、海关监管区、国家航空一级口岸等出口平台建设，积极争取国家级、省级汽车及零部件出口基地建设，大力支持双星东风、东风小康、平云工贸、湖北运银、房县聚达等企业开拓国际市场。加强与友好城市的交流合作，主动开展与京津冀受水区对口协作。

（四）以绿色发展为特色，加强生态文明建设

牢固树立尊重自然、顺应自然、保护自然的生态文明理念，把生态文明建设放在突出地位，融入经济、政治、文化、社会建设各方面和全过程，既求经济跨越，更要绿水青山，推进绿色发展，构造绿色GDP，努力建设美丽十堰。

——强化生态建设和环境保护。加快中英生态城市合作项目建设，大力争取南水北调中线水源保护生态补偿资金及相关政策，加快实施生态环保、生态农业、生态旅游等示范项目，继续抓好退耕还林、长防林等重点生态工程，完成人工造林30万亩。加强森林资源保护，提高森林覆盖率，创建国家森林城市。严禁违法开山。抓好饮用水水源保护，保障饮水安全。开展汉江库岸及消落区生态环境综合整治和地质灾害防治。加快神定河、泗河、犟河、官山河、剑河流域污染整治，打造水清、河畅、岸绿、景美的水污染防治示范工程。

——推进污染减排和资源节约管理。建立污染减排联席会议制度，加快淘汰高耗能、高污染行业落后产能。完善城镇环保基础设施，大力推进水泥厂、燃煤电厂脱硝工程，强化重金属、危险废物等污染物防控，新建污水管网200公里。强化大气污染防治，启动机动车尾气污染治理，形成PM2.5监测能力。推进循环经济发展和清洁生产，建设循环经济工业园和再生资源回收利用体系，切实提高资源利用效率。

——深化生态文明创建和制度建设。严格按照创模规划和方案要求，明确年度工作目标，扎实推进国家环保模范城市创建。开展生态乡镇（村）创建工作，争取创建国家级生态乡镇（村）2个、省级4个，推进绿色社区、绿色家庭、绿色学校等创建活动。研究制定有利于生态发展的政策措施，探索建立体现生态文明的目标体系、考核办法、奖惩机制。

（五）以保障和改善民生为根本，努力建设幸福十堰

强化民生GDP理念，坚持把保障和改善民生作为一切工作的根本出发点和落脚点，守住底线、突出重点、完善制度，倾注更多的精力、集中更多的财力解决民生问题，提高人民幸福指数，建设幸福十堰。

——完善基本公共服务体系。落实保障教育事业发展的各项政策，统筹各类教育协调发展，全面完成《学前教育三年行动计划（2011—2013年）》，基本解决入园难问题；推进中小学标准化建设，改造中小学危房6万平方米；推进“联校协作发展”工程，着力提高素质教育水平；市、区共同努力，三年内基本解决当前城区义务教育发展存在的突出问题；大力发展职业教育，积极支持高校发展。实施人才培养工程，加强人才队伍建设。进一步健全公共卫生服务体系，完善突发公共卫生事件应急和重大疾病防控机制，健全农村三级医疗卫生服务网络和城市社区卫生服务体系；积极探索新农合与居民医保的整合及市级统筹，健全城乡居民大病保障机制，新农合参合率稳定在95%以上；支持太和医院门诊综合大楼和太极湖院区、人民医院医技住院大楼、东风公司总医院第二临床学院、妇幼保健院新院选址建设。继续稳定低生育水平，综合治理出生人口性别比，提升计生优质服务水平。

——提高就业和社会保障水平。坚持稳定岗位与扩大就业并重，实施更加积极的就业政策，推动实现更高质量的就业。进一步扩大社会保险覆盖面，确保“五项保险”参保人数稳定在204.5万人；城乡低保标准分别提高20%和25%；切实保障低收入群体的基本生活，认真落实农村五保供养政策，做到“应保尽保、按标施保”。推进国办和社会化养老机构建设，改扩建农村福利院；提高医疗救助核销比例和临时救助标准。注重解决农民工市民化待遇，保障进城农民与城镇居民享有平等的权利。做好南水北调内安移民安稳致富工作，加快制定丹江口库区移民后续帮扶规划。加大住房保障力度，扩大公积金制度受惠人群。

——切实增加城乡居民收入。按照2020年城乡居民人均收入比2010年翻一番的目标，制订居民收入倍增计划。深化企业和机关事业单位工资制度改革，推行企业工资集体协商制度，多渠道增加居民财产性收入，力争农村和城镇居民收入分别增加595元和2080元。

——加强和创新社会管理。加强基层社会管理和服务体系建设，推广社区网格化服务管理，增强城乡社区服务功能。完善和创新流动人口和特殊人群管理服务，促进流动人口有序融入城镇、特殊人群顺利融入社会。积极排查化解矛盾纠纷，认真开展稳定风险评估和人民调解，加强和改进信访工作。深化平安十堰建设，完善立体化社会治安防控体系。完善食品安全监管体制，强化监测评估、检验检测，全面提高食品安全保障水平。高度重视安全生产，坚决防范重特大事故发生。加强全民国防教育，抓好国防动员和民兵预备役工作。深入开展双拥共建，巩固发展军政军民团结。

（六）以铸造美好精神家园为方向，切实抓好精神文明和民主法制建设

——繁荣发展文化事业。完善公共文化服务体系，启动各县市“两馆”建设，提升图书馆、美术馆、文化馆免费开放服务水平；继续实施广播电视“村村通”工程，启动“户户通”工程，支持发展无线移动广播电视和“十堰城市电视”。推进公共文体设施建设，开工建设十堰大剧院、传媒大厦、广电大厦、南水北调博物馆，推进体育公园二期工程，新建农民体育健身工程100个。积极开展文艺下乡，加强对世界文化遗产和非物质文化遗产保护，弘扬优秀传统文化。加速培育影视制作、时尚设计等文化产业，增强地方文化整体实力和竞争力。加强文化市场综合执法，强化网络文化建设和管理，规范文化市场秩序。

——深化精神文明创建。广泛开展社会公德、职业道德、家庭美德、个人品德教育，形成知荣辱、讲正气、促和谐的良好社会风尚。深化“十星级”系列文明创建活动，做好争创2011—2013年度全国、省级文明城市工作，精心组织迎接2013年全国、全省城市文明程度指数测评，力争实现全国文明城市提名资格“两连冠”和省级文明城市“七连冠”，确保新一轮全国、省级文明城市创建顺利申报。加强“诚信十堰”、“诚信社会”、“诚信政府”建设，增强全社会诚实守信意识。加强文明单位、文明家庭、文明市民等基础创建工作，不断提升社会文明素养。

——加强民主法制建设。自觉接受人大法律监督和政协民主监督。加强与群团组织联系，建立健全民主办事程序，深入实施“六五”普法，加强法治政府建设，提高依法行政水平。抓好反腐败和源头治腐工作，切实履行“一岗双责”。深入开展民主评议政风行风活动，坚决纠正损害群众利益的不正之风。加大大案要案查处力度，以反腐倡廉实际成效取信于民。

切实做好外事、侨务、对台工作，继续做好民族宗教、审计、统计、气象、地震、消防等工作。

（七）以加强政府自身建设为保障，着力提升工作效能

认真贯彻落实中央改进工作作风、密切联系群众的八项规定，切实加强政府自身建设，着力建好两支队伍。一是建设一支朝气蓬勃、求真务实，有浓厚宗旨观念、深受群众拥护的公务员队伍；二是建设一支有强烈事业心、高度责任感，政令畅通、落实有力、率先垂范、廉洁高效的政务领导干部队伍，做到“善谋事、会创新、能落实、敢担当”，铸就“善政、勤政、明政、严政、廉政、实政”的人民满意政府。

政府工作人员要有深厚的民本意识、良好的精神状态、严谨的科学态度、务实的工作作风、严明的职业纪律。深厚的民本意识就是密切同人民群众的血肉联系，把群众情绪作为“第一信号”，把群众满意作为“第一追求”，把群众向往作为“第一目标”。心中装着群众，感情贴近群众，行动服从群众，政策交给群众，决策尊重群众，工作深入群众，难事请教群众，政务亮给群众，创业依靠群众，认真倾听群众呼声，体察群众疾苦，心系群众冷暖，扎扎实实为民办事，真心实意替民解忧，使政府各项工作都有广泛的群众基础；良好的精神状态就是有强烈的事业心和高度的责任感，思想解放，正能量充足，抢抓机遇而不坐失良机，开拓进取而不因循守旧，善于自加压力、自我突破，有一股子闯劲、冲劲、韧劲，不辞辛劳、不避疲劳、不惧苦劳，敢于作为、勇于担当，夙夜在公、奋发向上；严谨的科学态度就是尊重科学，按市场经济规律、自然规律和社会发展规律办事，不蛮干、不瞎指挥，在作出重大决策前，认真做好调研论证，不断深化对市情、县情、乡情的认识，准确把握世情、国情、省情的变化，提高工作的科学化水平，真正成为政府工作的行家里手，把每项工作都抓在点子上，把每件事情办得更完美；务实的工作作风就是说实话、办实事、求实效，言必行、行必果，不做表面文章，不摆花架子，不玩空手道，形成耻于空谈、羞于作秀、勤奋务实、躬身实干、狠抓落实的浓厚氛围，真正做到深入实际、了解实情，多谋实干、善谋实干，工作扎扎实实、效果实实在在，坚决克服官僚主义、形式主义，增强政府的公信力；严明的职业纪律就是严格执行党和国家的法律、法规、制度要求，坚持原则，遵守规矩，严肃政风政纪，严格要求，严格教育，严格管理，加大治庸问责力度，坚决反对贪污腐败，坚决制止有令不行、有禁不止的行为，确保步调一致、政令畅通。大力压减会议和文件，摆脱文山会海束缚，减少不必要的应酬，把主要精力放在抓重大决策的落实上、抓实际问题的解决上。加强调查研究，在实践中不断完善工作举措，牢牢把握工作主动权。坚持和发扬艰苦奋斗、崇尚节俭的优良传统，坚决反对大手大脚、奢侈浪费的不良倾向，加大为基层、为企业服务的工作力度，切实提高政府工作效能。

在这里，我代表市政府向全市人民承诺：加强政府自身建设、转变工作作风从我做起，从市政府领导班子做起，从每一位政府组成人员做起，要求别人做到的自己先做到，要求别人不做的自己坚决不做。

各位代表！为人民群众办好事、办实事是政府应尽的职责。今年将继续为全市人民办好十件实事：一是完成通村公路养护7500公里；二是开展青年创业培训8000人，新增就业3万人；三是城区新建环保公厕5座以上；四是完成农村育龄妇女免费检查“两癌”8万人，孕前健康检查4万人；五是建设保障性住房16000套；六是改造城区义务教育学校D级危房1.3万平方米，新建中小学校3所；七是建设20个社区文化活动室；八是全面改造农村电压低的电网；九是中心城区启动建设5处以上立体停车场；十是启动市殡仪服务中心建设。

各位代表！奋进的号角已经吹响，跨越的征程已经迈步。让我们紧密团结起来，在市委的坚强领导下，解放思想、凝神竞进、奋勇争先、砥砺前行，用辛勤的汗水、百倍的努力，为加快区域性中心城市建设、全面建成小康社会而努力奋斗！
